# Supplementary material for: Composition of Fatty Acids in Bone Marrow of Red Deer from Various Ecosystems and Different Categories
Source: Molecules. 2022 Apr 13;27(8):2511. doi: 10.3390/molecules27082511 (PMC9027502; doi:10.3390/molecules27082511)
Supplement: Supplementary file 1 [file molecules-27-02511-s001.zip › molecules-1648348-supplementary.pdf]

molecules

Supplementary Materials

Composition of Fatty Acids in Bone Marrow of Red Deer from Various Ecosystems and Different Categories

Żaneta Steiner-Bogdaszewska [1], Katarzyna Tajchman [2,*], Piotr Domaradzki [3,*] and Mariusz Florek [3]

1 Institute of Parasitology of the Polish Academy of Sciences, Research Station in Kosewo Górne, 11-700 Mrągowo, Poland; kosewopan@kosewopan.pl (Z.S-B.)
2 Department of Animal Ethology and Wildlife Management, Faculty of Animal Sciences and Bioeconomy, University of Life Sciences in Lublin, Akademicka 13, 20-950 Lublin, Poland
3 Department of Quality Assessment and Processing of Animal Products, University of Life Sciences in Lublin, Akademicka 13, 20-950 Lublin, Poland; mariusz.florek@up.lublin.pl (M.F.)
* Correspondence: katarzyna.tajchman@up.lublin.pl (K.T); piotr.domaradzki@up.lublin.pl (P.D.)

Table S1. Composition of Josera Phosphoreimer multi-ingredient licks (Josera, Poland).

Components		Content (per 1 kg)
Ca	%	5.00
P	%	10.00
Na	%	7.00
Mg	%	7.50
Ca / P		0.5:1
Vitamin A	j.m.	650 000.00
Vitamin D3	j.m.	120 000.00
Vitamin E	mg	1 500.00
Zn (as zinc oxide)	mg	8 000.00
Mn (as manganese chelate of glycine hydrate)	mg	4 000.00
Mn (as manganese (II) oxide)	mg	4 000.00
Cu (as copper sulphate pentahydrate)	mg	1 200.00
I	mg	100.00
Co	mg	22.00
Se (as sodium selenite)	mg	40.00
